# Exploring Canine Picornavirus Diversity in the USA Using Wastewater Surveillance: From High-Throughput Genomic Sequencing to Immuno-Informatics and Capsid Structure Modeling

**DOI:** 10.3390/v16081188

**Published:** 2024-07-24

**Authors:** Temitope O. C. Faleye, Peter Skidmore, Amir Elyaderani, Sangeet Adhikari, Nicole Kaiser, Abriana Smith, Allan Yanez, Tyler Perleberg, Erin M. Driver, Rolf U. Halden, Arvind Varsani, Matthew Scotch

**Affiliations:** 1 Biodesign Center for Environmental Health Engineering, Biodesign Institute, Arizona State University, Tempe, AZ 85287, USA; 2 College of Health Solutions, Arizona State University, Tempe, AZ 85287, USA; 3 School of Sustainable Engineering and the Built Environment, Arizona State University, Tempe, AZ 85287, USA; 4 Biodesign Center for Fundamental and Applied Microbiomics, Center for Evolution and Medicine, School of Life Sciences, Arizona State University, Tempe, AZ 85287, USA; arvind.varsani@asu.edu

**Keywords:** wastewater targeted sequencing, genotype-to-serotype prediction, *Picornaviridae*, immuno-informatics, virus capsid structure prediction

## Abstract

The SARS-CoV-2 pandemic resulted in a scale-up of viral genomic surveillance globally. However, the wet lab constraints (economic, infrastructural, and personnel) of translating novel virus variant sequence information to meaningful immunological and structural insights that are valuable for the development of broadly acting countermeasures (especially for emerging and re-emerging viruses) remain a challenge in many resource-limited settings. Here, we describe a workflow that couples wastewater surveillance, high-throughput sequencing, phylogenetics, immuno-informatics, and virus capsid structure modeling for the genotype-to-serotype characterization of uncultivated picornavirus sequences identified in wastewater. Specifically, we analyzed canine picornaviruses (CanPVs), which are uncultivated and yet-to-be-assigned members of the family *Picornaviridae* that cause systemic infections in canines. We analyzed 118 archived (stored at −20 °C) wastewater (WW) samples representing a population of ~700,000 persons in southwest USA between October 2019 to March 2020 and October 2020 to March 2021. Samples were pooled into 12 two-liter volumes by month, partitioned (into filter-trapped solids [FTSs] and filtrates) using 450 nm membrane filters, and subsequently concentrated to 2 mL (1000×) using 10,000 Da MW cutoff centrifugal filters. The 24 concentrates were subjected to RNA extraction, CanPV complete capsid single-contig RT-PCR, Illumina sequencing, phylogenetics, immuno-informatics, and structure prediction. We detected CanPVs in 58.3% (14/24) of the samples generated 13,824,046 trimmed Illumina reads and 27 CanPV contigs. Phylogenetic and pairwise identity analyses showed eight CanPV genotypes (intragenotype divergence <14%) belonging to four clusters, with intracluster divergence of <20%. Similarity analysis, immuno-informatics, and virus protomer and capsid structure prediction suggested that the four clusters were likely distinct serological types, with predicted cluster-distinguishing B-cell epitopes clustered in the northern and southern rims of the canyon surrounding the 5-fold axis of symmetry. Our approach allows forgenotype-to-serotype characterization of uncultivated picornavirus sequences by coupling phylogenetics, immuno-informatics, and virus capsid structure prediction. This consequently bypasses a major wet lab-associated bottleneck, thereby allowing resource-limited settings to leapfrog from wastewater-sourced genomic data to valuable immunological insights necessary for the development of prophylaxis and other mitigation measures.

## 1. Introduction

The SARS-CoV-2 pandemic increased the scale of viral genomic surveillance globally [1]. However, in resource-limited settings (RLSs), one of the barriers to transitioning this development into meaningful immunological insight that is valuable for the development of countermeasures like diagnostics, vaccines, and chemotherapeutic agents is the wet lab constraints (economic, infrastructural, and personnel). One potential avenue for RLSs to leapfrog this barrier is to explore bioinformatics workflows that couple phylogenetics, immuno-informatics, and virus structure (and capsid) prediction/modeling for the genotype-to-serotype characterization of uncultivated viruses whose genomes have been determined. Furthermore, coupling the abovementioned with wastewater (WW) surveillance can facilitate the incorporation of WW surveillance benefits like One Health potential, low cost, population-wide insights, and early warning capacity while still ensuring that data generated can feed directly into the development of medical countermeasures. Here, we demonstrate the utility of the abovementioned using an uncultivated picornavirus.

Canine picornavirus (CanPV) is a yet-to-be-assigned and uncultivated member of the subfamily *Ensavirinae* in the family *Picornaviridae*. CanPVs have a ~8 kb positive-sense, single-stranded RNA genome that encodes one large polyprotein, flanked on both ends by untranslated regions (UTRs). CanPVs have been identified in the feces, urine, respiratory swabs, and liver (suggesting they might be capable of causing systemic infection) of dogs and red foxes in the United Arab Emirates (UAE), China, Hong Kong, Australia, and, more recently, in municipal WW in the USA [2,3,4,5,6,7]. Very little is known about CanPV sequence diversity, and, prior to this study, less than 20 sequences were publicly available in GenBank. Phylogenetic analysis of CanPV variants detected in WW in the USA suggested silent (and likely enzootic) circulation in the USA for over 15 years [4]. Despite this, there has been no documented detection or description of the virus in canines in the USA, which has over 70 million dogs [8] living in residential areas as pets or in shelters. Hence, there are unanswered questions about CanPV genotype diversity, genotype–serotype boundaries, epidemiology, host range, pathogenesis, and possible zoonotic potential that need urgent answers. One of the critical barriers to progress in CanPV research is understanding CanPV global diversity, which will facilitate definitive studies to elucidate genotype–serotype boundaries, epidemiology, and possible clinical manifestations.

In this study, we explore CanPV diversity using wastewater targeted sequencing (WTS). Considering CanPV prevalence of about 1% in dogs [7], thousands of samples would be required to document CanPV diversity in any population. However, our previous detection of CanPV presence in WW [3,4] and the ability of WTS to sample thousands to hundreds of thousands of individuals in a population simultaneously make it the best approach to investigate CanPV diversity on a population scale.

Here, we describe a pan-CanPV complete capsid gene amplification long-range reverse-transcriptase polymerase chain reaction (RT-PCR) assay, coupled with wastewater surveillance, high-throughput nucleotide sequencing, phylogenetics, immuno-informatics, and virus capsid structure modeling. Our results show that there is ongoing circulation of multiple CanPV genotypes in the USA and between continents. We also show at least eight genotypes that belong to four clusters, with intracluster divergence of <20%, and that our data suggest might be distinct serological types. Further exploration of CanPV is necessary to better understand its biology, evolutionary dynamics, diversity, and potential clinical manifestations, among other factors.

## 2. Materials and Methods

### 2.1. Sample Collection and Processing

Samples archived at −20 °C at the Human Health Observatory in Biodesign Institute, Arizona State University, Tempe, AZ, USA, were used in this study. This study utilized 118 wastewater samples collected from ten different sites in two municipalities (human population; ~700,000) in Maricopa County, Arizona (USA), between October 2019 and March 2020 (season 1) and October 2020 and March 2021 (season 2). Samples from all ten sites were from the same day of the month, except for in November 2020, when a sample from one site was collected within 24 h of the others. Also, only eight (8) sites were sampled in March 2020 because two of the locations could not be sampled for logistic reasons due to the onset of the SARS-CoV-2 pandemic. Each sample was collected over 24 h using time- or flow-weighted automated samplers.

**Figure 1:**
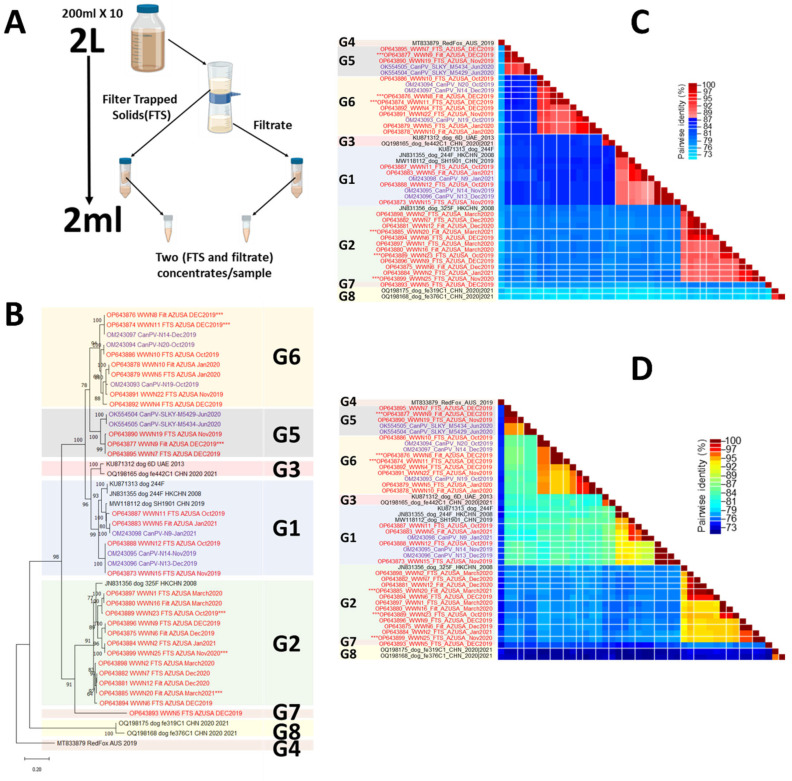
(**A**) Schematic representation of the wastewater processing workflow used in this study. (**B**–**D**) Genetic characterization of CanPV variants. (**B**) Maximum likelihood (ML) tree of CanPV variants present in GenBank as of June 2023 and those recovered in this study. The ML tree was inferred using IQ-Tree 1.6.12., with the best substitution model (TIM2 + F + I + G4) selected using ModelFinder. (**C**,**D**) Pairwise similarity analysis of CanPV variants in (**B**). (**C**) Cut-offs at >87% (i.e., <13% divergence) in 2 colors. (**D**) The same similarity profile in rainbow mode. Please note that sequences whose names are labeled with a red color were the ones generated in this study. Those with asterisks did not have the complete capsid sequence. Those in purple were previously described by our group and detected serendipitously while screening for enteroviruses. Those in black were described by others and downloaded from GenBank.

For each of the 12 months, the ten samples per month were recovered from a freezer and thawed overnight. Subsequently, 200 mL of wastewater from each of the ten sites was filtered using ten 450 nM membrane filters (Thermo Fisher Scientific, Waltham, MA, USA). The filtrates were pooled and concentrated to 2 mL (~1000× concentration) using 10,000 molecular weight (MW) cutoff centrifugal filters. The membrane filters were also recovered, and filter-trapped solids (FTSs) were resuspended by vortexing (Heidolph Instruments, Schwabach, Germany) for 10 min at 3000 rpm in a 50 mL centrifuge tube containing 25 mL of sterile PCR-grade water containing 15 glass beads (3 mm, Cole-Parmer, IL, USA). After vortexing, the filters were removed and the mixture was centrifuged for 20 min at 3900 rpm and 4 °C. The supernatant was recovered, pooled, and concentrated to 2 mL using 10,000 MW cutoff centrifugal filters (Figure 1A). Hence, for each of the 12 months, there were two concentrates, one for filtrate and one for FTS.

### 2.2. Nucleic Acid Extraction and Polymerase Chain Reaction (PCR)

All 24 concentrates were subjected to nucleic acid extraction using the QIAamp viral RNA MiniKit following the manufacturer’s instructions. The extract was used to screen for CanPV using a nested PCR assay (Tables S1 and S2). All assays were run using a BioRad 1000 thermal cycler (BioRad, Hercules, CA, USA). Second-round PCR amplicons were resolved on 2% agarose gels stained with GelRed (Biotium, Fremont, CA, USA) and viewed using the BioRad Gel Doc XR+ system running Image lab v4.1 software (BioRad, Hercules, CA, USA).

Since no CanPV complete capsid sequence amplification assay existed at the time this study started, CanPV sequences publicly available in GenBank as of 31 January 2022 were downloaded, aligned, and used to design primers for amplifying the complete capsid using Geneious Prime software v2022.0 [9]. The CanPV complete capsid sequence amplification RT-PCR assay amplified a ~3900 bp fragment of the genome encompassing the complete capsid protein-coding genomic region. Amplicons from the CanPV complete capsid sequence assay were used as a template for two second-round CanPV PCR assays targeting the VP2 (~250 bp) and VP2-VP3 (~950 bp) gene segments (Tables S1 and S2).

### 2.3. Sequencing

A random subset of amplicons generated from the second-round assays were cleaned and Sanger-sequenced using their respective forward and reverse primers. This was to confirm that the first-round assays worked and that the target virus (and genomic region) was amplified. Subsequently, the first-round amplicons of all samples positive for the second-round assays were cleaned and used for library preparation and paired-end sequencing (2 × 250 bp) on an Illumina MiSeq sequencer (Illumina, San Diego, CA, USA) at the Biodesign Institute, Arizona State University, USA.

### 2.4. Reads Processing

The Illumina raw reads were processed on the KBase platform using default parameters [10]. Specifically, raw reads were trimmed using Trimmomatic v.0.36. The trimmed reads were then *de novo* assembled using metaSPAdes v3.15.3. Contigs were identified using a BLASTn search of the GenBank database [11]. To confirm that variants found in both FTS and filtrates from the same WW sample were identical, trimmed reads from both fractions were merged and reassembled. Contigs that were present in the different fractions but that coalesced in the merged analysis were considered the same.

### 2.5. Virus Typing and Phylogenetic Analysis

Virus typing was performed using phylogenetic and pairwise identity analyses. Multiple sequence alignment (MSA) was performed using ClustalW in MEGA X [12], and a maximum likelihood tree was constructed with 1000 bootstrap replicates in IQ-Tree [13]. Prior to phylogenetic tree construction, the best-fitting nucleotide substitution model was selected using ModelFinder [14]. Pairwise identity was estimated using SDT v1.2. [15].

### 2.6. Immunoinformatics and CanPV Capsid Structure Prediction

B-cell epitopes present in VP1, VP2, and VP3 were predicted using Bepipred Linear Epitope Prediction 2.0 [16]. CanPV protomer prediction was performed using alphafold2 as implemented in ColabFold [17]. The complete particle was oligomerized from protomers using the oligomer generator available in Viperdb [18]. Structure annotation was performed using ChimeraX v1.8 [19].

## 3. Results

### 3.1. Nested PCR Results

We detected CanPVs in 58.3% (14/24) of the samples. Both nested PCR assays detected the same 14 samples as positive for CanPVs (Table 1). Both FTS and filtrates of February 2020 and 2021 and October 2020 (six samples in all) were negative for CanPV. The four remaining negative samples were filtrates from October 2019, November 2019, and November 2020 and FTS from March 2020 (Table 1).

### 3.2. Reads and Contigs Obtained

Post-trimming, we had 13,824,046 Illumina reads (Table S3) from the 14 samples, from which 27 CanPV contigs (Table S4) were assembled from 59.91% of the trimmed reads (Table S3). Contig length ranged from 2037 nt to 3906 nt and mean depth of coverage ranged from 2735× to 62,494× (Table S4).

### 3.3. Virus Typing

Phylogenetic analysis showed that the CanPV contigs detected in this study and genomes (complete or partial) publicly available in GenBank formed eight (8) distinct clusters with strong bootstrap support (Figure 1B). Pairwise identity analysis showed that divergence within each of the eight clusters was less than 13% (Figure 1C). However, these eight clusters formed four higher-order clusters, within which, divergence was less than 20% (Figure 1D).

**Figure 2:**
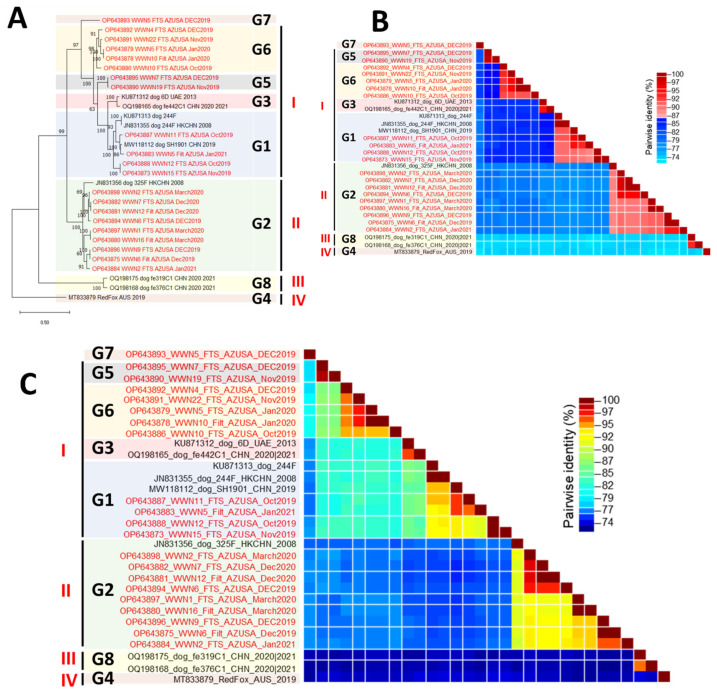
Genetic characterization of CanPV variants for which complete capsid (VP4-VP1) sequences were available. Please note that sequences in Figure 1 highlighted purple and those with triple asterisks were not included in this analysis because they did not contain the complete capsid sequence. (**A**) Maximum likelihood (ML) tree of complete capsid sequences of CanPV variants present in GenBank as of June 2023 and those recovered in this study. The ML tree was inferred using IQ-Tree 1.6.12., with the best substitution model (TIM2 + F + I + G4) selected using ModelFinder. (**B**,**C**) Pairwise similarity analysis of CanPV variants in (**A**). (**B**) Cut-offs at 86% (i.e., 14% divergence) in 2 colors. (**C**) The same similarity profile in rainbow mode.

**Figure 3:**
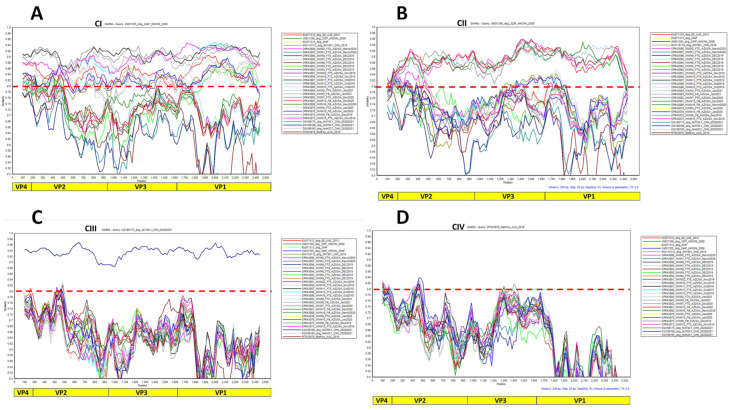
Simplot analysis showing the <20% diversity threshold for each cluster. In (**A**–**D**), a member of each of the clusters I, II, III, and IV, respectively, was selected as a query.

When contigs that did not cover the complete capsid were excluded from the analysis, phylogenetic analysis still showed the presence of eight (8) distinct clusters with strong bootstrap support (Figure 2A). Pairwise identity analysis, however, showed that divergence within each of the eight clusters was less than 14% (Figure 2B). These eight clusters still formed four higher-order clusters, within which, divergence was less than 20% (Figure 2C). Simplot analysis using members of each of the four higher-order clusters as query confirmed that divergence was less than 20% (Figure 3). Furthermore, pairwise identity analysis of VP1, VP2, and VP3 at both the nucleotide and amino acid levels confirmed the four-cluster architecture (Figure S1). In addition, it also showed that divergence was less than 20% at the nucleotide level and <10% at the amino acid level (Figure S1).

Note the fact that G7 demonstrated inconsistent clustering patterns in similarity analyses of VP1, VP2, and VP3 nucleotides and amino acids, respectively (Figure S1). Furthermore, Simplot analysis using G7 as a query showed divergence greater than 20% across P1, except in the VP1 genomic region, where it clustered with members of cluster I (Figure S2). Consequently, G7 is not classified in this discussion as part of any of the four clusters.

B-cell epitope prediction highlighted five epitopes distributed between VP1, VP2, and VP3 with signatures that were unique for members of each of the four clusters (Table 2). Interestingly, while these regions were variable between the four clusters, there was significant conservation within each cluster (Table 2).

**Figure 4:**
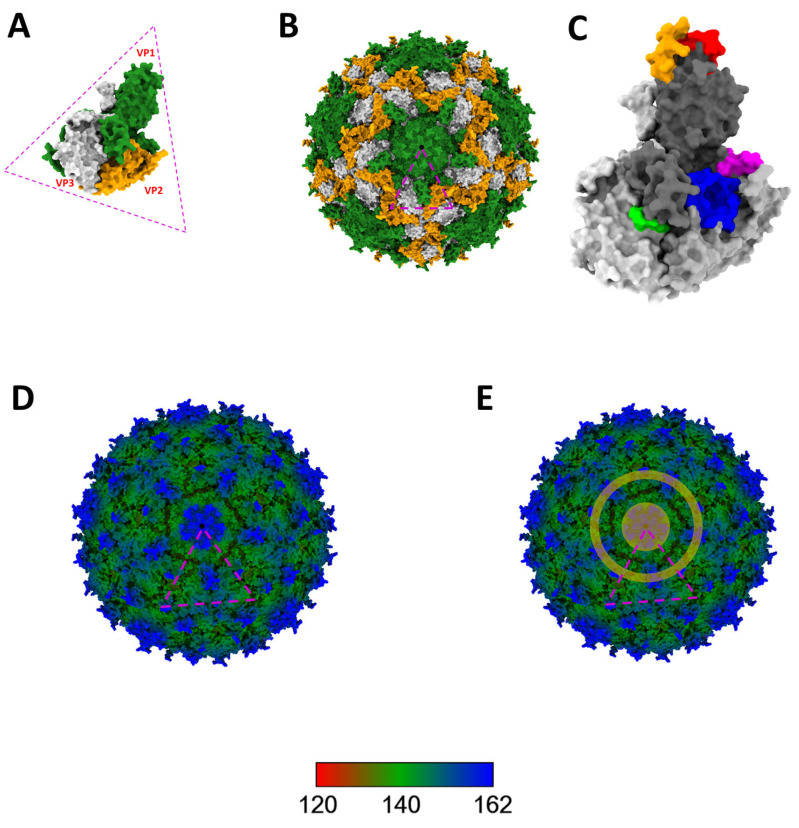
Predicted CanPV protomer and complete particle structure. (**A**) Predicted structure of CanPV protomer with VP1, VP2, and VP3 colored green, orange, and light grey, respectively. (**B**) Predicted capsid colored as detailed in A. (**C**) Protomer in A colored in different shades of grey and the predicted clusters of unique B-cell epitopes in Table 2 layered on it in different colors. (**D**,**E**) Predicted CanPV capsid structure radially colored to show the surface topology. In addition, E highlights (in orange) the distribution of the predicted B-cell epitopes around the northern and southern canyon walls.

Protomer structure prediction showed the juxtaposition of some of the predicted B-cell epitopes to form conformational epitopes (Figure 4C). Furthermore, our analysis showed the clustering of the predicted B-cell epitopes around the northern and southern rims of the canyon surrounding the five-fold axis of symmetry (Figure 4E).

### 3.4. Diversity by Fraction and Season

**Figure 5:**
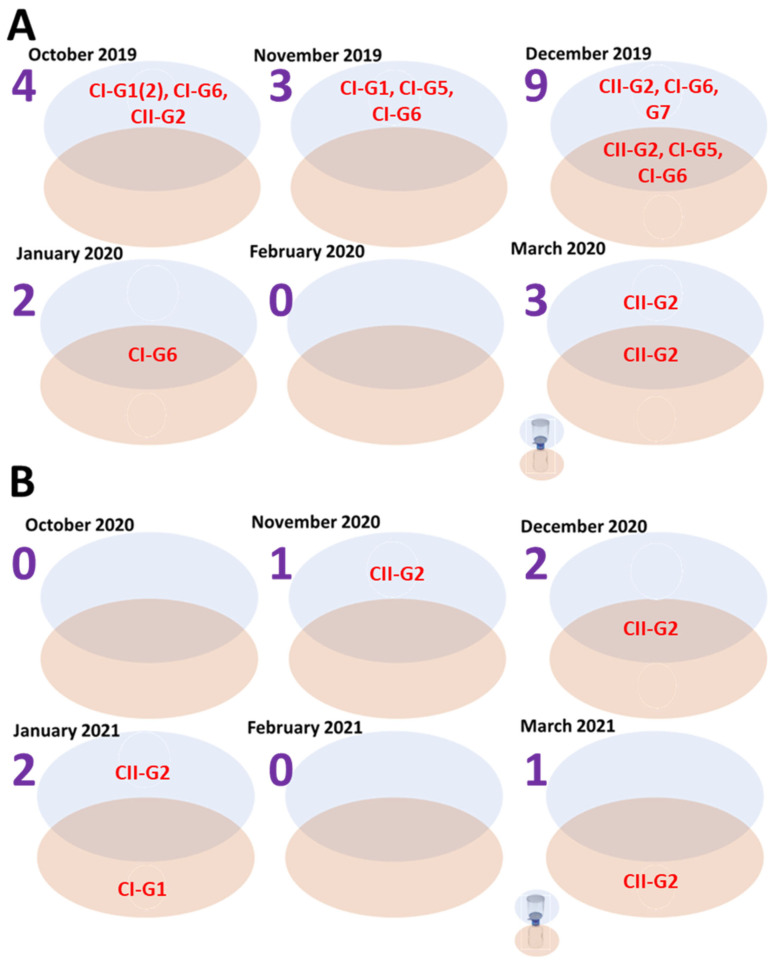
CanPV variants detected in different fractions, FTS (top, blue oval) and filtrate (bottom, beige oval), of WW from October 2019 to March 2020 (**A**) and October 2020 to March 2021 (**B**). Those detected in both are in the overlapping region of both ovals. The blue number beneath each month shows the number of variants detected per month.

Overall, only members of clusters I and II were identified from WW in AZ, USA, in this study. Specifically, for season 1, 21 CanPV variants (belonging to two clusters and five genotypes) were detected, 16 from FTS (from five genotypes) and 5 from filtrates (from three genotypes). All three CanPV genotypes detected from filtrates were also detected in FTS. However, two CanPV genotypes (G1 and G7) were detected in only FTS, while none was uniquely detected in filtrates. In summary, for season 1, CanPV genotype and variant diversity detected in FTS were consistent with what was found in filtrates, but the genotype and variant diversity detected in the filtrates were not consistent with what was found in FTS. However, variants belonging to both clusters I and II were detected in both FTS and filtrates (Figure 5 and Figure S3).

For season 2, six CanPV variants (belonging to two genotypes and both clusters I and II) were detected, three each from FTS and filtrates. Genotype G2 was detected in both FTS and filtrates. However, genotype G1 was detected in filtrates but not in FTS. In December 2020, the same CanPV variant was detected in both FTS and filtrates. For the other months (except for October 2020 and February 2021, where both fractions were negative), different CanPV variants were detected in both fractions. In summary, for season 2, CanPV type and diversity detected in FTS were not consistent with what was detected in the filtrates, and vice versa (Figure 5 and Figure S3).

A 3.5× decrease in CanPV variants was detected between seasons 1 and 2. Twenty-one and six CanPV variants were detected in seasons 1 and 2, respectively. Only two (G1 and G2) of the five CanPV types detected in season 1 were detected in season 2. The remaining three CanPV genotypes (G5, G6, and G7) were not detected in season 2 (Figure S3).

## 4. Discussion

Though there have been previous reports [2,3,4,5,6,7] describing the detection of CanPV genomes in various sources, there has been no attempt to genotype or ‘serotype’ these uncultivated viruses. In this study, phylogenetic and pairwise identity analyses showed eight (8) distinct CanPV genotypes with strong bootstrap support. Divergence within each genotype was less than 14% (Figure 2). These genotypes, however, formed four clusters, with intracluster divergence of <20% (Figure 3 and Figure S1), and which our results suggest are distinct serological types. Specifically, similarity analysis, immuno-informatics, and CanPV protomer and capsid structure prediction showed the aggregation of cluster-distinguishing B-cell epitopes (Table 2) in the northern and southern rims of the canyon surrounding the 5-fold axis of symmetry of the predicted CanPV capsid (Figure 4). These findings are consistent with well-established principles in enterovirus type designation and serotyping [20].

The best-studied picornaviruses are those in the genus *Enterovirus* (EV) in subfamily *Ensavirinae* (to which CanPVs belong). Within this genus, genotype designation was originally based on serotypes defined by neutralization assays [21,22]. It has been shown that EV-neutralizing antibodies work by either (1) binding the three-fold axis of symmetry and consequently causing conformational changes that result in the capsid releasing the genomic cargo or by (2) sterically occluding binding of the receptor to the receptor binding site (which is usually within the canyon surrounding the 5-fold axis of symmetry) on the virus by binding to epitopes around (northern or southern canyon rims) or that overlap the receptor binding site [20]. Furthermore, studies [21,22] demonstrated a correlation between serotypes and nucleotide sequences in the genomic region encoding capsid proteins. Consequently, serotyping has been replaced by pairwise identity in the capsid region and members of the same ‘serotype’ have divergence ranging from less than 25% for Enterovirus A to less than 12% for Rhinovirus B. Therefore, for CanPV, the aggregation of cluster-distinguishing B-cell epitopes in the northern and southern rims of the canyon surrounding the 5-fold axis of symmetry coupled with divergence of <20% fits well within the *Enterovirus* (and possibly *Ensavirinae*)-type demarcation paradigm.

It is not clear what the intracluster genotypes (<14% divergence) present in cluster I indicate. However, we think these CanPV genotypes (lineages) might suggest something fundamental in the biology of CanPV and might have some implications for their molecular epidemiology. Specifically, we consider them to be similar to the lineages of any enterovirus type like Enterovirus A71 lineages A to F [23] or Echovirus 30 lineages [24,25]. It is important to note that our data suggest that there is circulation of multiple CanPV genotypes (and likely serotypes) in the USA. Our data also suggest that CanPVs might be circulating between continents (Figure 1 and Figure 2). Furthermore, considering at least four lineages with intra-lineage divergence of <14% already documented for cluster I, it is highly likely that clusters II, III, and IV are currently under-sampled (Figure 1 and Figure 2).

We found a decrease in both virus presence and diversity between seasons 1 and 2 (Figure 5 and Figure S3). It is not clear whether this difference between seasons may reflect the impact of nonpharmaceutical interventions used to mitigate the spread of SARS-CoV-2 in 2020/2021. If so, virus type and variant diversity data generated from WW surveillance in this study might have captured the impact of population-wide changes in human behavior in response to the SARS-CoV-2 pandemic and how this has impacted the dynamics of viruses (and possibly other microorganisms) in non-human animals in municipalities. Whether this trend (a drop in variant detection and diversity) has been observed with domesticated animal infectious agents remains to be seen.

It is important to mention that, while outside the USA, CanPV has been detected in dogs and red foxes [2,5,6,7]; in the USA, it has only been detected in WW [3,4]. Hence, though CanPV is circulating in the US, we do not know its host(s) in the country. However, we have preliminary data (unpublished) showing an abundance of canine parvoviruses in the WW concentrates analyzed in this study. The conundrum, however, is that, generally, in the population sampled, pet owners oversee outdoor defecation by their dogs, pick up the feces in plastic bags, and, subsequently, dispose of these in public trash cans, whose contents end up in landfills. Hence, dog feces (in this locality) generally end up in landfills rather than in WW. Furthermore, the stormwater collection system is distinct from the wastewater system in the geographical location sampled in this study. This therefore reduces the likelihood that the WW samples could have been contaminated by runoff from the streets and sidewalks. On the other hand, there is ample research [26] describing canine parvovirus detection in domestic and wild cats, and flushing cat litter into the sewer system is common practice in the community (personal communication). It is therefore likely that the canine parvoviruses we are detecting in the WW might be largely of feline origin and puts cats on the list of suspect reservoirs of CanPV in Arizona, USA. Therefore, we currently do not know the source of the CanPVs detected in municipal WW in the population sampled in this study. However, our findings highlight the significance of WW genomic surveillance for a One-Health approach to pathogen surveillance.

In this study, we also considered whether size fractionation of WW samples prior to ultrafiltration impacts our perception of virus presence and diversity in WW samples, and our results suggest that it might (Figure 5 and Figure S3). Specifically, not all samples had congruence between types and diversity in FTS and filtrates. For example, while CanPV was detected in FTS in November 2020, no virus was detected in the corresponding filtrate. Hence, with respect to virus presence or absence in the WW sample, both (FTS and filtrate) partition-based results are inconsistent with each other (Table 1 and Figure 5 and Figure S3). Previous studies [27,28,29,30,31] investigating the impact of sample clarification on virus presence assumed that virus presence and diversity are one and the same. Hence, they did not examine how the clarification process impacts our perception of virus diversity in both fractions. In this study, our results show the importance of using both partitions for a rigorous and robust evaluation of virus diversity during WW surveillance. For example, while CanPV was in both partitions in January 2021, G2 and G1 were uniquely detected in the FTS and filtrate, respectively (Figure 5). Furthermore, when summed over the course of either one or both seasons (Figure S3), our data showed that virus types could be present in a population and WW clarification by size fractionation could determine whether it is detected over the course of a six-month period. Such a delay in virus variant detection could undermine the early warning function of WW surveillance by delaying our ability to detect variants circulating in the population.

The abundance of large, particulate fecal matter in the primary clarification chamber of wastewater treatment plants (WWTPs) shows that not all fecal matter in WW completely dissolves and release trapped virus particles into suspension. Hence, the possibility exists that some of the viruses recovered in FTS might have been those trapped in undissolved fecal matter or even adsorbed to solids in WW. Additionally, it has been shown that enteric bacteria are adept at aggregating enteric viruses on their surfaces [32,33,34] and, in the process, stabilizing these virions and increasing their ‘resistance’ to inactivation under harsh conditions. Since the clarification process aggregates bacteria, it might consequently further aggregate enteric viruses that are already on bacterial surfaces. Taken together with the results of this study, it seems clear that WW clarification by size fractionation might be removing viruses from the filtrate and, consequently, the final concentrate may lose some virus diversity. This further emphasizes the need to ensure that viruses are recovered from both FTS and filtrates for a more representative description of virus presence and diversity in any population.

There is an increased need for quick and reliable ways to glean insights that are valuable for public health decision-making and the development of countermeasures from the wealth of genomic data now publicly available, courtesy the availability and accessibility of multiple high-throughput sequencing platforms. As a response to this, here, we describe a workflow that couples wastewater surveillance, high-throughput sequencing, phylogenetics, immuno-informatics, and virus capsid structure prediction for the genotype-to-serotype characterization of uncultivated picornavirus genomes (using canine picornaviruses as a case study). This approach bypasses significant wet lab constraints (economic, infrastructural, and personnel) of translating novel variant or sequence detection in wastewater (or other matrices) to meaningful immunological and structural insights that are valuable for the development of vaccines, diagnostics, and other mitigation measures (especially for emerging and re-emerging viruses). This workflow therefore allows resource-limited settings to leapfrog from genomic data recovered from wastewater (or other pathogen genomic surveillance sources) to valuable immunological and structural insights necessary for the development of countermeasures.

The limitations of this study include our use of samples that were archived at −20 °C. It is possible that the freeze–thawing process could have resulted in a reduction in virus titer. If association with bacteria protects particle integrity during the freeze–thaw process as it does to heat inactivation and bleach [34], then this could be partly responsible for the difference in CanPV detection between FTS and filtrates. Furthermore, a long-range PCR assay was used for our first-round PCR assay. Hence, we might have missed fragmented genomes that might have been detected by assays targeting smaller genomic regions or real-time RT-PCR assays. Studies are therefore needed replicating the experiments described here with fresh, unfrozen samples. Also, we understand that the CanPV diversity described here (both from our study and in GenBank) might not be representative of global population-level diversity. Hence, there is a need for more studies to better document CanPV global diversity.

Genotype G7 highlights another limitation of this study. Figure S1 shows that different regions of the G7 capsid sequence clustered with different sequences, suggesting that it might be a recombinant. Furthermore, Figure S2 shows that the VP4, VP2, and VP3 regions of G7 followed the 80% rule (suggesting the existence of another cluster). However, Figures S1 and S2 both show that the VP1 region clustered with members of Cluster I. While G7 might be a recombinant, there is also the possibility that it could be an artifact of the workflow (short-read sequencing coupled with *de novo* assembly) employed in this study. This G7 and the fact that recombination contributes significantly to the evolution of picornaviruses (though it is more commonly detected in the non-capsid genomic regions of these genomes) highlights the need for workflows in WW surveillance that couple long-range RT-PCR with long-read sequencing. In fact, it would better serve WW surveillance if we developed long-range RT-PCR workflows that recover complete capsid and/or complete genomes from WW as a single contig and couple such with long-read sequencing. This will not only enable better resolution of contigs like G7 but it will also make it possible to explore the evolutionary dynamics of both the capsid and non-capsid genomic regions of such viruses from genomes recovered from WW, without the need for growing these viruses in cell culture. As proof of concept, we have successfully developed such a workflow for CanPV [35]. Such systems will be beneficial for the surveillance of many viruses of medical importance (like vaccine-derived polioviruses), for which global restrictions are in the pipeline on growing them in culture in nonessential facilities [36,37].

## Figures and Tables

**Table 1:** Concentrates analyzed in this study and virus detection by nested PCR. Please note that both nested PCR assays detected the same 14 samples as positive for CanPVs. ‘+’ indicates that a virus was detected.

Month	Filtrate		FTS	
	Conc ID	CanPV	Conc ID	CanPV
October 2019	1		13	+
November 2019	2		14	+
December 2019	3	+	15	+
January 2020	4	+	16	+
February 2020	5		17	
March 2020	6	+	18	+
	Season 1	3/6		5/6
October 2020	7		19	
November 2020	8		20	+
December 2020	9	+	21	+
January 2021	10	+	22	+
February 2021	11		23	
March 2021	12	+	24	
	Season 2	3/6		3/6
	Total	6/12		8/12

**Table 2:** B-cell epitope prediction results detailing the major cluster-defining epitopes in VP2, VP3, and VP1, respectively, and their location relative to the canyon around the 5-fold axis of symmetry of the predicted capsid structure (please see Figure 4). Amino acid numbering is relative to OP643895.1, conserved residues are in bold font, and amino acid variants within the cluster are noted by “/”.

	VP2	VP3	VP1		
Phylogenetic Clusters	South-Wall Rim (aa 194–217)	South-Wall Rim (aa 56–62)	North-Wall Rim (aa 143–161)	South-Wall Rim (aa 219–228)	North-Wall Rim (aa 246–254)
C-I	**CQ**SSSGE**R**S/LS/NT**IF**E**L**A/V/TD/EN/T/I/Y**DF**R/G**DYP**	SN/SD/EGNV	**DP**V**S**RARN/DQV/IPNIS**D**	Q**NF**S/N**P**S/TAA/VG**Y**	SYRTNDGV**S**
C-II	**CQ**SSSGE**R**SN/DS**IF**Q/P**L**QQER**DF**A/S**DYP**	SD/ENTNTF	**DP**A**S**RSRNNVPNLT**D**	R**NF**N**P**EA/TAG**Y**	NFRSSDNT**S**
C-III	**CQ**AS/PQA**R**TSS**IF**E**L**QDT**DF**Q**DYP**	SGEVT/ATP	**DP**S/T**S**RNRVNGS/NA/TTIPNNS**D**	Q**NF**Q**P**QTSG**Y**	SYKDNNT**S**
C-IV	**CQ**SSSGE**R**SAS**IF**Q**L**TEQ**DF**A**DYP**	GESATP	**DP**S**S**TGLSNSTTIS**D**	A**NF**Q**P**QAAQ**Y**	ARKDNTGT**S**

## Data Availability

The sequences described in this study have been deposited in SRA and GenBank under accession numbers PRJNA892620 and OP643873–OP643899, respectively.

## Supplementary Materials

The following supporting information can be downloaded at https://www.mdpi.com/article/10.3390/v16081188/s1: Table S1: Reverse-transcriptase polymerase chain reaction (RT-PCR) and PCR reaction conditions for assays used in this study. Note that assay 1 was performed using SuperScript™ III One-Step RT-PCR System with Platinum™ Taq DNA Polymerase and assays 2 and 3 were performed using GoTaq green PCR master mix. See Table 2 for details of the primer sequence. Table S2: Primers used in this study. See Table 1 for details of reaction conditions. Numbers represent genomic locations of primer binding sites (PBSs) in MW118112. Please note that, in our hands, these primers were compatible with the addition of GC clamps (for other downstream workflows) to the 5’ends without impacting the amplification of the target sequence. Table S3: Summary of Illumina raw reads generated, trimmed, and mapped to CanPV contigs in this study. FTS = filter-trapped solids. Table S4: Mean coverage depth of CanPV contigs detected in this study. Cells in ‘red’ denote complete capsid not recovered post-assembly. Table S5: Global distribution of CanPV types described to date based on sequence data publicly available in GenBank as of February 2023. Note that members of each genotype have divergence below 14%. Please see Figure 2 for genotype classification. Figure S1: CanPV classification based on VP1, VP2, and VP3 nucleotide and amino acid sequence diversity. A, C, and E and B, D, and F show nucleotide and amino acid divergence, respectively. Figure S2: Simplot analysis showing the peculiarity of G7. Notice compliance with the ~20% diversity threshold from VP4 to VP3 but breach of the rule in VP1. Topology suggests either a recombination event or an artifact of the de novo assembly. However, this demonstrates that there might be other ‘serotypes’ or clusters currently undescribed that obey the ~20% diversity threshold. Figure S3: CanPV diversity detected in each season and fraction. Variants detected in FTS or filtrate are clustered in blue or beige rectangles, respectively. Red-colored variants are those unique to a fraction within each season.
