# Supplementary material for: Exploring Canine Picornavirus Diversity in the USA Using Wastewater Surveillance: From High-Throughput Genomic Sequencing to Immuno-Informatics and Capsid Structure Modeling
Source: Viruses. 2024 Jul 24;16(8):1188. doi: 10.3390/v16081188 (PMC11359023; doi:10.3390/v16081188)
Supplement: Supplementary file 1 [file viruses-16-01188-s001.zip › viruses-3041415-supplementary.pdf]

Supplementary File

Table S1: Reverse-transcriptase polymerase chain reaction (RT-PCR) and PCR reaction conditions for assays used in this study. Note that assay 1 was done using SuperScript™ III One-Step RT-PCR System with Platinum™ Taq DNA Polymerase and assays 2 and 3 were done using GoTaq green PCR master mix. See Table 2 for details of primer sequence.

	Region amplified	Amplicon size (bp)	RT	Pre-heat	# of cycles	Denaturation	Annealing	extension	Final incubation
Assay 1	Complete capsid	~3900	50ºC – 30 min	94ºC – 2 min	42x	94ºC – 15 sec	55ºC – 30 sec	68ºC – 8 min	68ºC – 5 min
Assay 2	Nested partial VP2	~250	N/A	94ºC – 2 min	35x	94ºC – 15 sec	55ºC – 30 sec	60ºC – 30 sec	68ºC – 5 min
Assay 3	Nested partial VP2-VP3	~950	N/A	94ºC – 2 min	35x	94ºC – 15 sec	55ºC – 30 sec	60ºC – 60 sec	68ºC – 5 min

Table S2: Primers used in this study. See Table 1 for details of reaction conditions. Numbers represent genomic location of primer binding site (PBS) in MW118112. Please note that in our hands these primers are compatible with the addition of GC-clamps (for other downstream workflows) to the 5'ends without impacting amplification of the target sequence.

	Primer Name	Primer Sequence (5'-3')
Assay 1	CPV-443F	CTTCAGTAATCCTCCGGCCC
	CPV-4321R	GCAATGAGCACAATGGCACAAATTG
Assay 2	CPV-1089F	GCATATGGGGAATGGCCTGA
	CPV-1309R	ACACAAAAACCAGATCGCATGA
Assay 3	CPV-1089F	GCATATGGGGAATGGCCTGA
	CPV-2023R	TTTGAAAAGAACCTTGTGCATCT

Table S3: Summary of Illumina raw reads generated, trimmed, and mapped to CanPV contigs in this study. FTS = Filter-trapped-solids.

	Month-Year	Conc-ID	Total # Raw reads	Total # trimmed reads	Total # mapped reads	Total # mapped reads (%)
Filtrate	Dec-19	3	930,978	929,244	630,397	67.84
Filtrate	Jan-20	4	1,048,618	1,045,692	628,945	60.15
Filtrate	Mar-20	6	1,021,308	1,018,062	618,284	60.73
Filtrate	Dec-20	9	410,080	407,816	160,034	39.24
Filtrate	Jan-21	10	890,000	887,056	523,488	59.01
Filtrate	Mar-21	12	971,240	967,016	98,642	10.2
FTS	Oct-19	13	1,146,120	1,143,542	801,737	70.11
FTS	Nov-19	14	1,168,766	1,163,982	329,132	28.28
FTS	Dec-19	15	1,000,374	998,860	913,946	91.5
FTS	Jan-20	16	1,118,184	1,116,248	940,890	84.29
FTS	Mar-20	18	1,671,848	1,668,836	1,498,275	89.78
FTS	Nov-20	20	787,894	784,834	63,052	8.03
FTS	Dec-20	21	826,726	823,142	658,421	79.99
FTS	Jan-21	22	872,380	869,716	416,873	47.93
		**Total**	13,864,516	13,824,046	8,282,116	59.91

Table S4: Mean coverage depth of CanPV contigs detected in this study. Cells in 'red' denote complete capsid not recovered post-assembly.

S/N	Accession	Collection Month/Year	FTS/Filt	Contig length [nt]	mean coverage	Complete Capsid		Type	Cluster
1	OP643886	Oct/2019	FTS	3,524	10,428x	Yes		G6	I
2	OP643887	Oct/2019	FTS	3,471	13,100x	Yes		G1	I
3	OP643888	Oct/2019	FTS	3,168	18,600x	Yes		G1	I
4	OP643889	Oct/2019	FTS	2,037	4,192x	No		G2	II
5	OP643873	Nov/2019	FTS	3,891	6,176x	Yes		G1	I
6	OP643890	Nov/2019	FTS	3,548	5,491x	Yes		G5	I
7	OP643891	Nov/2019	FTS	3,186	5,056x	Yes		G6	I
8	OP643874	Dec/2019	FTS	2,173	19,737x	No		G6	I
9	OP643892	Dec/2019	FTS	3,551	4,322x	Yes		G6	I
10	OP643893	Dec/2019	FTS	3,536	12,129x	Yes		G7	?
11	OP643894	Dec/2019	FTS	3,530	2,735x	Yes		G2	II
12	OP643895	Dec/2019	FTS	3,448	3,024x	Yes		G5	I
13	OP643896	Dec/2019	FTS	2,767	11,917x	Yes		G2	II
14	OP643875	Dec/2019	Filt	3,044	11,140x	Yes		G2	II
15	OP643876	Dec/2019	Filt	2,761	21,807x	No		G6	I
16	OP643877	Dec/2019	Filt	2,676	10,316x	No		G5	I
17	OP643879	Jan/2020	FTS	3,900	44,319x	Yes		G6	I
18	OP643878	Jan/2020	Filt	3,906	30,773x	Yes		G6	I
19	OP643897	Mar/2020	FTS	3,572	9,559x	Yes		G2	II
20	OP643898	Mar/2020	FTS	3,551	62,494x	Yes		G2	II
21	OP643880	Mar/2020	Filt	3,910	29,811x	Yes		G2	II
22	OP643899	Nov/2020	FTS	2,843	4,053x	No		**G2***	**II**
23	OP643882	Dec/2020	FTS	3,883	29,191x	Yes		G2	II
24	OP643881	Dec/2020	Filt	3,891	7,772x	Yes		G2	II
25	OP643884	Jan/2021	FTS	3,901	19,826x	Yes		G2	II
26	OP643883	Jan/2021	Filt	3,898	25,341x	Yes		G1	I
27	OP643885	Mar/2021	Filt	2,178	7,348x	No		G2	II

Table S5: Global distribution of CanPV types described to date based on sequence data publicly available in GenBank as of February 2023. Note that members of each genotype have divergence below 14%. Please see Figure 2 for genotype classification.

	SOURCES				
**Genotype**	**WW**	**Dogs**			**Red Foxes**
	USA	Hong Kong	China	United Arab Emirates	Australia
G1	2019, 2021	2008	2019		
G2	2019, 2020, 2021	2008			
G3			2020-2021	2013	
G4					2019
G5	2019, 2020				
G6	2019, 2020				
G7	2019				
G8			2020-2021		

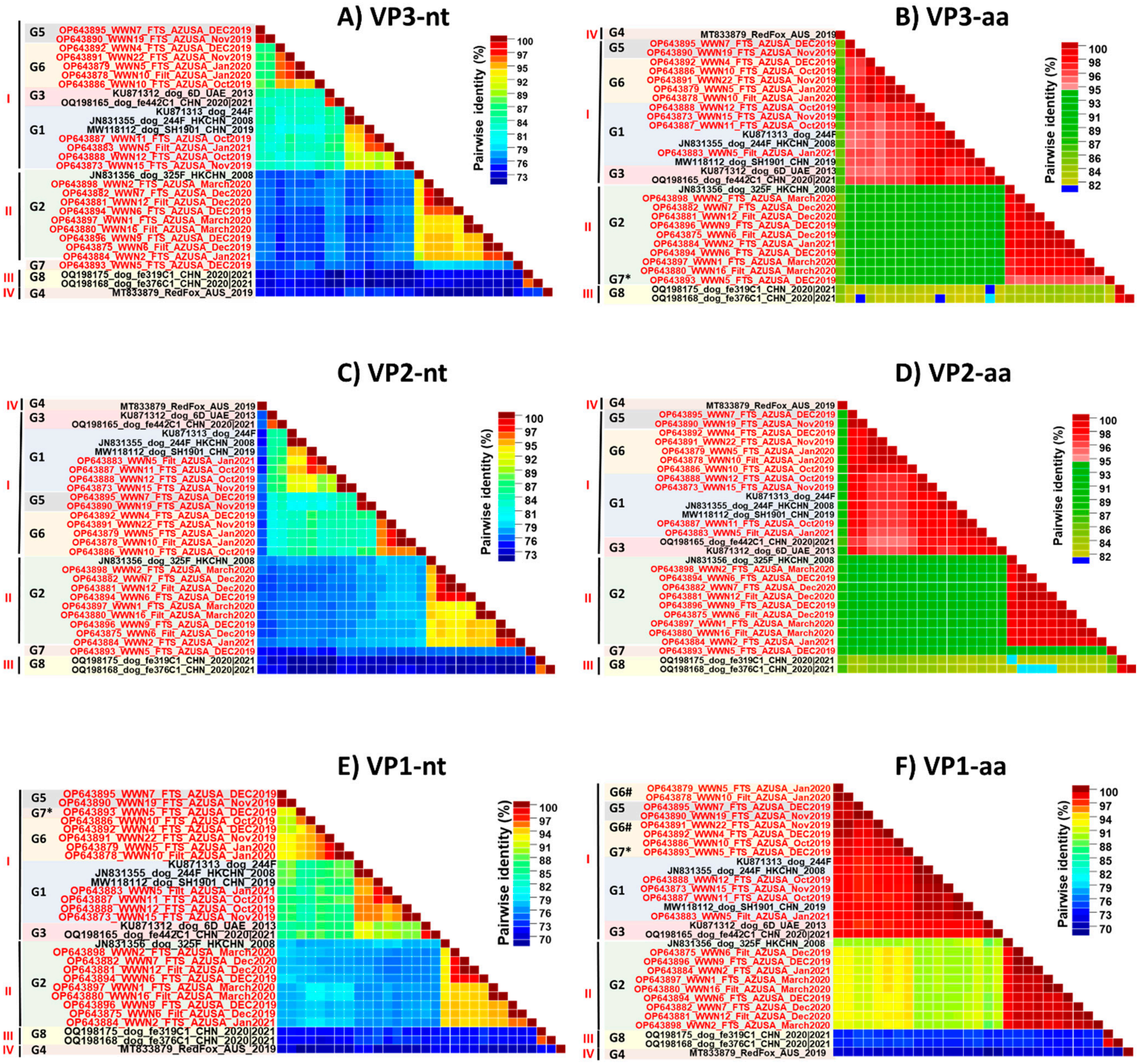

Figure S1: CanPV classification based on VP1, VP2 and VP3 nucleotide and amino acid sequence diversity. A, C, E and B, D, F show nucleotide and amino acid divergence respectively.

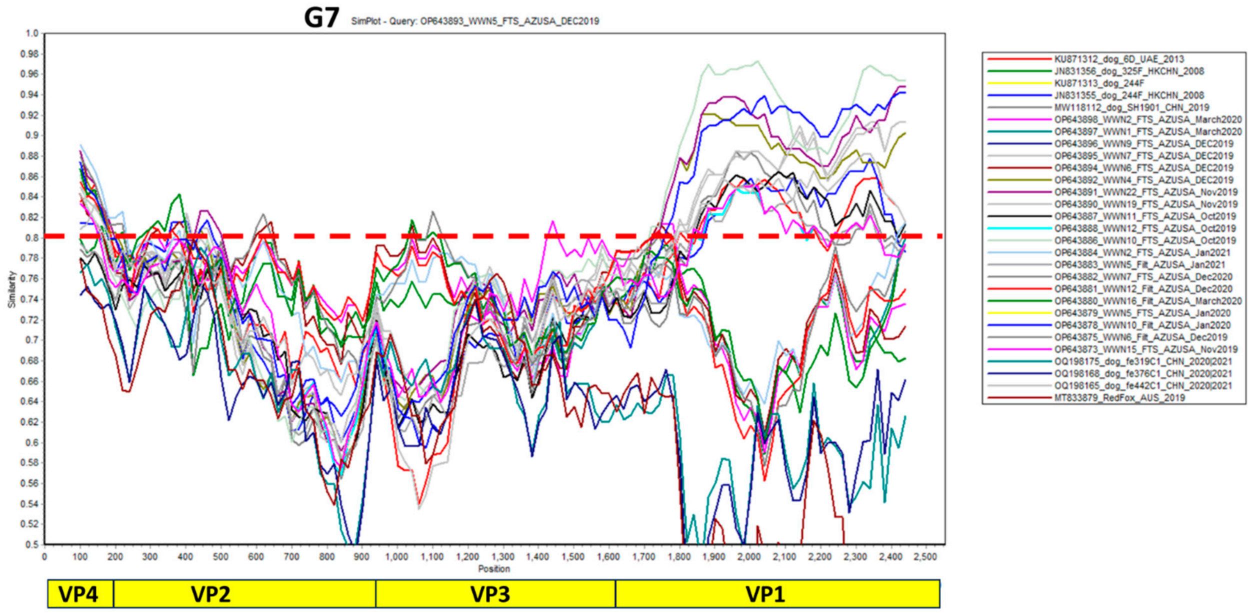

Figure S2: Simplot analysis showing the peculiarity of G7. Notice compliance with the ~20% diversity threshold from VP4 to VP3 but breach of the rule in VP1. Topology suggests either a recombination event or artifact of the *de novo* assembly. However, it demonstrates there might be other "serotypes" or clusters currently undescribed that obey the ~20% diversity threshold.

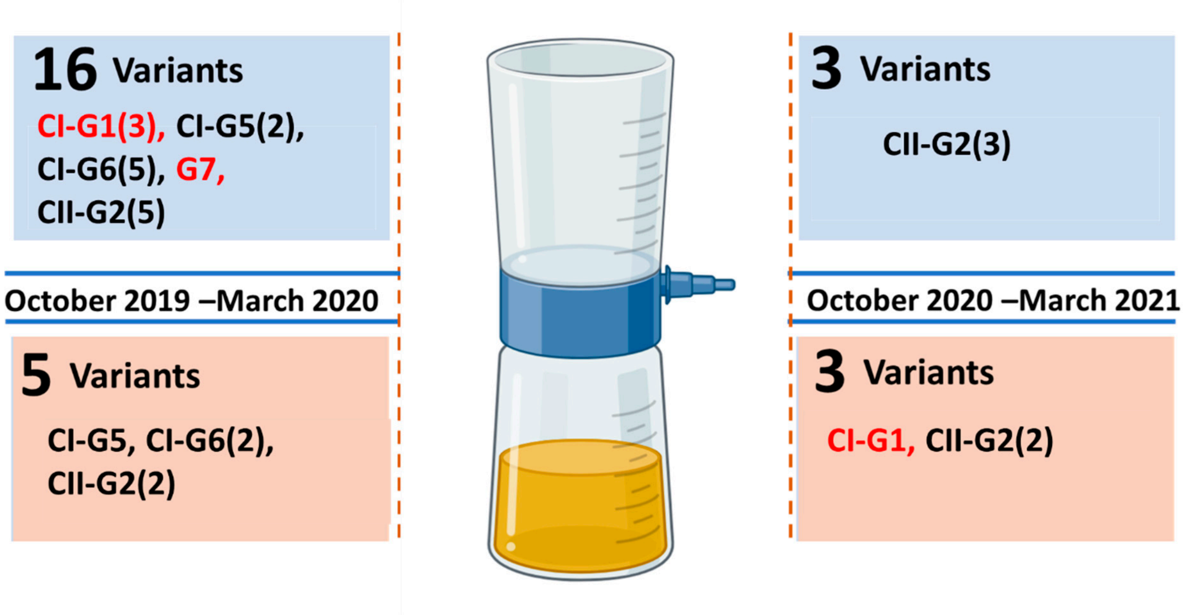

Figure S3: CanPV diversity detected in each season and fraction. Variants detected in FTS or filtrate are clustered in blue or beige rectangles, respectively. Red colored variants are those unique to a fraction within each season.
